# Template-Based Geometric Simulation of Flexible Frameworks

**DOI:** 10.3390/ma5030415

**Published:** 2012-03-12

**Authors:** Stephen A. Wells, Asel Sartbaeva

**Affiliations:** 1 Department of Physics, University of Warwick, Gibbet Hill Lane, Coventry, CV4 7AL, UK; 2 Inorganic Chemistry Laboratory, Department of Chemistry, University of Oxford, South Parks Road, Oxford, OX1 3QR, UK; E-Mail: asel.sartbaeva@chem.ox.ac.uk

**Keywords:** framework, rigid unit, geometric simulation, flexibility, zeolite, perovskite

## Abstract

Specialised modelling and simulation methods implementing simplified physical models are valuable generators of insight. Template-based geometric simulation is a specialised method for modelling flexible framework structures made up of rigid units. We review the background, development and implementation of the method, and its applications to the study of framework materials such as zeolites and perovskites. The “flexibility window” property of zeolite frameworks is a particularly significant discovery made using geometric simulation. Software implementing geometric simulation of framework materials, “GASP”, is freely available to researchers.

## 1. Introduction

Template-based geometric simulation is a specialised method for the study of flexible frameworks, which we may define as follows: *a flexible framework is a structure composed of relatively rigid subunits connected by relatively flexible linkages*. By *relatively rigid* and *relatively flexible*, we mean that the energetic penalty for distortions of the subunits is significantly greater, e.g., by at least an order of magnitude, than the energetic penalty for variations in the geometry of the linkage. In the context of materials, the classic example of such a structure is a three-dimensional framework silicate, either dense (e.g., quartz) or porous (e.g., a zeolite). Here the relatively rigid subunit is the SiO${}_{4}$ tetrahedron, while the relatively flexible linkage is the Si–O–Si bridge. A wide range of Si–O–Si angles are observed in different framework silicate structures [1,2], indicating that the bridging angle can be varied over a substantial range without excessive energetic penalty.

The existence of two disparate energy scales for distortions of the framework naturally suggests an idealisation, generally known as the Rigid Unit model [3,4]. In this idealisation, the subunits are treated as entirely rigid. Constraints on the linkages are then chosen to maintain the connectivity of the structure while allowing free angular variation. For a framework silicate, a rigid unit model considers each SiO${}_{4}$ unit as a tetrahedron, with a harmonic constraint of zero natural length connecting the corners of linked tetrahedron. Since this model allows, at some energy penalty, for the corners of linked tetrahedra not to be colocated, it can also be termed a “split-atom” model. This model has been implemented in the “CRUSH” software [5] for the prediction in reciprocal space of Rigid Unit Modes or RUMS—low-frequency collective modes, involving cooperative rotations of polyhedra, which are significant in dynamic disorder and as soft modes for phase transitions [6].

In contrast, template-based geometric simulation combines insights from both the atomic and polyhedral views of a structure. This method maintains two *overlapping* representations of the structure. In the atomic representation, all atoms are present and have coordinates and radii for the detection of contact steric interactions. In addition, a polyhedral template is superimposed on each polyhedral unit; this template is sometimes referred to as a “ghost”. The interatomic bonding within the unit is represented by harmonic constraints linking each atom to a vertex of the template [7]. These constraints penalise deviations of the atomic geometry in the unit from the ideal geometry represented by the “ghost” template. This use of templates to represent the bonding geometry of multiple atoms is the most distinctive feature of template-based geometric simulation; conventional simulations using empirical interatomic potentials would make use of multiple two-body (bond distance) and three-body (bond angle) terms. Bridging atoms belonging to multiple polyhedral units are linked to one vertex on the template for each unit.

**Figure 1:**
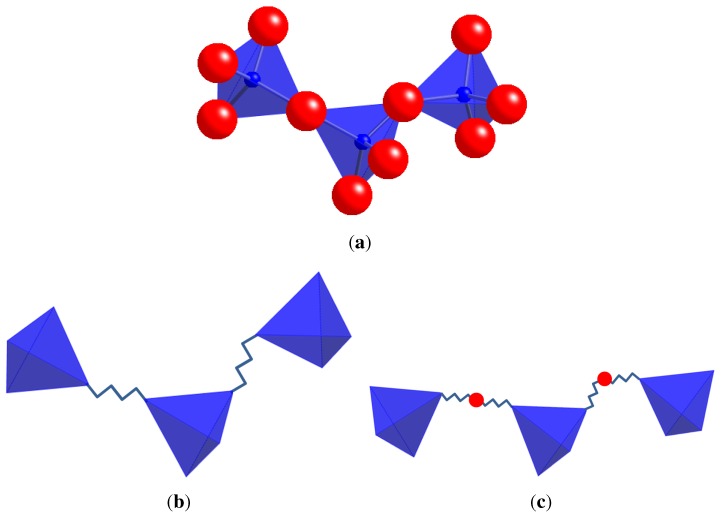
(**a**) Part of a silicate mineral framework containing Si and O atoms, with the SiO${}_{4}$ units viewed as polyhedra; (**b**) a rigid-unit model of the framework for calculation of RUMs. The polyhedra are considered as rigid units and their vertices are connected by harmonic constraints; (**c**) templates and constraints for geometric simulation. A tetrahedral template is centred on each Si atom. Vertex O atoms are tethered by harmonic constraints to template vertices.

The development of the rigid unit model and of template-based geometric simulation from the polyhedral view of a framework is illustrated in Figure 1.

As we shall see, this template-based approach lends itself to both the geometric analysis of structural models and to the generation of new structural models by geometric simulation. The method is implemented primarily in a piece of software titled “GASP”, for “Geometric Analysis of Structural Polyhedra”, which is freely available to researchers and can be obtained from the corresponding author. The name “GASP” was intended as a respectful pun on the widely used and extremely comprehensive “GULP” simulation package [8].

## 2. Comparison to Conventional Simulation Methods

It is instructive to consider both the limitations and the advantages of such a specialised and physically simplified method of simulation. The method is of course on an entirely different level of theory from *ab-initio* electronic structure methods, and indeed from all-atom empirical-potential methods, which seek to generate accurate structures and generate realistic energy landscapes. Geometric simulation does not generally include any long-range forces and essentially neglects electrostatics. The production of detailed energetics is not the goal.

The method has some attractive computational features. It handles large system sizes well, it is robust and it gives sensible results for framework geometry without requiring, for example, detailed knowledge of the distribution of extra-framework content. The main advantage of a simplified simulation, however, is in the generation of insight. Since geometric simulation includes geometric effects while excluding charge, polarity and other long-range effect, it can indicate whether such long-range effects are required to explain a given framework behaviour. By focussing on real-space rigid unit behaviour, the method suggests and answers questions that other methods would not; the most important example being the discovery of the “flexibility window” phenomenon in zeolite frameworks (Section 4.6).

If our goal is to produce very accurate energy landscapes for a structure, then we require the highest level of theory that is available to us. For the generation of insight, however, the most detailed model is in a sense the least useful, since what it tells us is that a very accurate model of the real system behaves as the real system does. The value of simplified methods lies in the deliberate inclusion of only certain parts of the physics and the exclusion of others. If the simplified model generates a behaviour which we wish to explain in a real system, then we have learned something about what aspects of the physics are important in producing that behaviour. For this reason the development of simplified models and methods will always form a parallel strand to the ongoing development of more and more accurate *ab-initio* and empirical-potential methods.

## 3. Templates for Geometric Analysis and Simulation

A basic item of input for geometric analysis and simulation is one or more *polyhedral specifications*, giving the elements to be found at the centre and at the vertices of the polyhedron, the polyhedral shape (from a small list of defined shapes), and the ideal bond length. The combination of shape and bond length specifies the shape of the polyhedral template to be matched to a bonded group of atoms. The principal shapes used in geometric simulation to date have been tetrahedra and octahedra; for example an SiO${}_{4}$ tetrahedron is typically specified as “TET Si O 1.61”, while a regular MnO${}_{6}$ octahedron would be specified as “OCT Mn O 1.9” [9]. On the basis of these specifications every atom in a structure can be assigned a role by element, as centre, vertex or “interstitial” if it is not part of any polyhedral type.

The structural model is provided to the simulation in .xtl format in P1 symmetry, that is, with all atoms explicitly represented. Bonded groups of atoms in the structural model can be found either by specifying the bond connectivity *a priori* or using a distance-based search centred on each central atom. A geometrically regular polyhedron of appropriate type is then constructed over each bonded group and each atom in the group is assigned a vertex on the polyhedron. After the construction process, the position and orientation of the template must be aligned to the positions of the atoms in its group.

### 3.1. Polyhedral Alignment and Residual Mismatch

At the heart of the geometric analysis and simulation approach is a system for efficiently matching the position and orientation of two similar polyhedra so that the total mismatch between the positions of their vertices is minimised. The residual mismatch then represents the deformation of one polyhedron relative to the other.

A convenient representation for rotations in three dimensions is found in the bivector algebra of geometric or Clifford algebra, and the use of geometric algebra “rotor” operators in the alignment process is described in [10]; hence the use of “geometric simulation” as a description of the method.

This polyhedral alignment is used to align the geometrically regular template polyhedra to the, generally slightly irregular, polyhedra formed from the positions of atoms in a bonded group. For frameworks we typically centre the template on the central atom and then minimize the mismatches for the vertex atoms with respect to the orientation of the template. The residual mismatch then represents deformations of the geometry of the bonded group away from the geometric ideal defined by the template.

**Figure 2:**
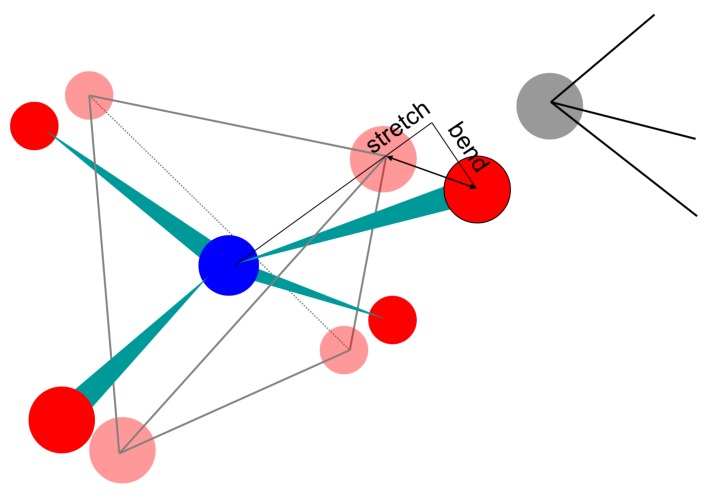
The mismatch between an atom and a template vertex is decomposed into components of bond-stretching, parallel to the bond vector in the template, and of bond-bending, perpendicular to the bond vector in the template.

An immediate effect of using a template is to provide a useful single measure of polyhedral deformation, with units of length, which can be decomposed into bond-stretching and bond-bending terms. The vector describing the mismatch between an atomic position and a template vertex can be resolved into components parallel and perpendicular to the vector describing the centre-vertex bond in the template. The parallel component represents bond stretching or compression, and the perpendicular component represents deformation of the internal angles of the polyhedron. One can thus quantify the distortion of a polyhedron in terms of the RMS bond-stretching and bond-bending mismatch among its vertices. This is particularly valuable in making angular deformations of polyhedra immediately understandable and comparable to variations in bond length; such deformations would otherwise have to be reported as a list of internal (vertex-centre-vertex) angles. The decomposition of distortions into bending and stretching components is illustrated in Figure 2.

It is not necessary for one of the polyhedra in the alignment to be geometrically regular and one can also consider the alignment and residual mismatch of two irregular polyhedra from different structural models. This provides means to quantify the relative importance of rigid-unit motion and polyhedral distortion in the dynamic or static disorder of frameworks (Section 4.1), and to examine polyhedral rotation in the response of a framework to pressure (Section 4.3).

This single-step alignment of polyhedra—either fitting templates to a model or comparing two models—constitutes geometric *analysis* of the polyhedral framework. To proceed to a geometric *simulation* we must also develop a force model based on the mismatches between atoms and templates, as discussed in Section 3.2.

### 3.2. Force Models in Geometric Simulation

The force model for geometric simulation consists of harmonic constraints of zero natural length penalising the mismatch between atoms and templates, plus harmonic (soft-sphere) constraints penalising steric overlap of atoms, considered as spheres. Steric clashes between atoms belonging to the same bonded group are not considered.

**Figure 3:**
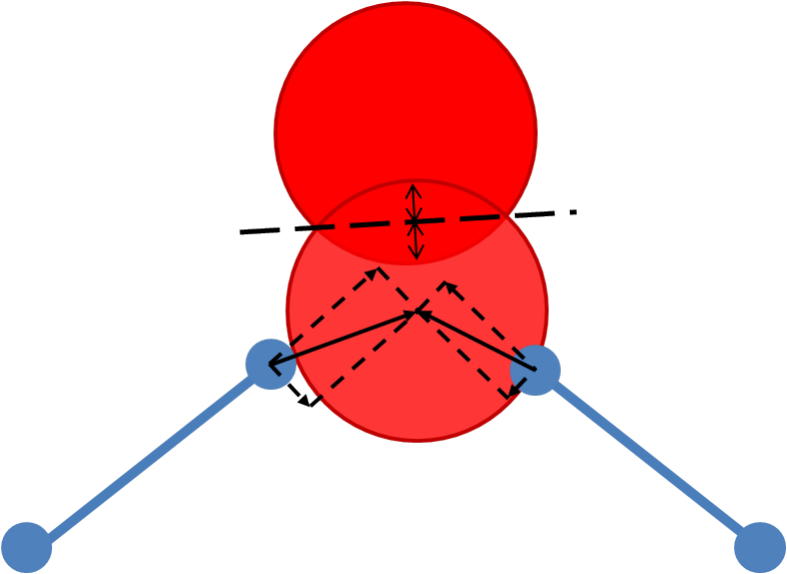
Force model for geometric simulation. The oxygen atom at the centre of the diagram is in steric contact with a nearby atom (above) and is connected to two template vertices to right and left. The mismatch of the atom from a template position is resolved into bond-stretching and bond bending components. Harmonic penalties apply to the bond-bending distortions, bond-stretching distortions, and steric overlap of atomic spheres.

As the mismatches can be resolved into bond-bending and bond-stretching components, these components can be assigned separate harmonic constraints and need not have the same spring constant. The simulation calls for the specification of spring constants for bond-bending and bond-stretching for each polyhedral type. In the method’s most common application, to silicate zeolite frameworks, we typically assign values of 5 and 20 arbitrary units as the spring constants for bond-bending and bond-stretching distortions of the SiO${}_{4}$ tetrahedra, based on the relative frequencies of the corresponding modes of vibration observed experimentally in SiO${}_{2}$ structures [11,12,13]. The simulation also requires a steric radius for each element present, and a spring constant. We typically treat steric clashes as being as disadvantageous as bond stretching and assign a spring constant of 20 arbitrary units to all overlaps. The most significant radius for zeolite frameworks is that of the tetrahedral oxygen atoms, for which we use a standard radius of 1.35Å [14]. The elements of the force model for geometric simulation are illustrated in Figure 3.

So long as the simulation includes only these terms—mismatch and steric overlap—the overall energy scale can be considered arbitrary. It is, however, possible to include other interactions in the simulation [12] using more conventional forms of interatomic potential, such as a Buckingham potential. In this case the energy scale for the geometric simulation terms and the other potentials must be sensibly matched (see Section 4.2).

A “geometric relaxation” of a framework attempts to minimise the penalties on mismatches and steric clashes with respect to (i) the positions of all the atoms and (ii) the positions and orientation of the templates. The approach taken in GASP is iterative. A polyhedral alignment step as described in Section 3.1 is followed by an atomic relaxation step in which the templates remain fixed while the atoms are moved by a simple steepest-descent algorithm. Polyhedral and atomic steps are repeated alternately until the penalty is considered sufficiently small or no further improvement is possible. Since this approach typically relaxes even large structures—supercells containing hundreds or thousands of atoms—in a few CPU-minutes we have not found it necessary to use more sophisticated minimisation algorithms. An experimental version of GASP making use of the limited-memory BFGS algorithm has been implemented by Kapko et al. [15,16].

## 4. Applications of Geometric Analysis and Simulation

In this section we shall briefly review the main applications so far of geometric analysis and simulation by the current authors and others. We shall consider the analysis of dynamic disorder (Section 4.1), the importance of framework flexibility for local motion (Section 4.2), compression mechanisms in zeolites (Section 4.3), the generation of hypothetical zeolite frameworks (Section 4.4), studies on regular and Jahn–Teller distorted manganite perovskites (Section 4.5), the “flexibility window” property of zeolite frameworks (Section 4.6), pressure-induced phase transitions in zeolites (Section 4.7) and the application of geometric simulation in biophysics for the simulation of flexible motion in protein (Section 4.8).

### 4.1. Dynamic Disorder in Frameworks

Geometric analysis is particularly useful in the analysis of dynamic disorder in polyhedral frameworks. Let us suppose that we have two or more structural models of a framework, representing “snapshots” of dynamic disorder, and that the models have the same framework topology, so that each polyhedron in one model can be matched to its equivalent in the other. Geometric analysis of a single model, matching templates to the model polyhedra, quantifies the distortions of the polyhedra from the geometric ideal. Geometric analysis of a pair of models, meanwhile, matches pairs of model polyhedra and decomposes their differences into components of rotation and distortion. This allows a quantitative assessment of the relative importance of rigid-unit motions and distortions in the dynamic disorder.

An ideal source of such structural snapshots is Reverse Monte Carlo modelling based on total neutron scattering data [17,18,19]. This method generates large structural models based on both sharp (Bragg) and diffuse scattering, so that the models represent both the average structure and the dynamic disorder about that average. Two independent models generated by fitting to the same data set represent uncorrelated snapshots of dynamic disorder. GASP was originally developed to analyse such structural models for SiO${}_{2}$ quartz in its α and β phases [7]. This analysis revealed a substantial component of rigid-unit motion in the dynamic disorder of quartz. This rigid-unit motion component displays strong temperature dependence with classic tricritical phase-transition behaviour, whereas the polyhedral distortion is almost constant with temperature and insensitive to the α–β phase transition.

Such structural modelling and analysis of framework silicates has revealed that the hexagonally symmetric β phase of quartz is a dynamic average, and that the local structure is more similar to the trigonally symmetric structure of α quartz. Molecular-dynamics investigations by Kimuzuka et al. have confirmed that the elastic behaviour of β quartz cannot be accounted for on the basis of the average structure [20].

This combination of Reverse Monte Carlo modelling and geometric analysis—RMC+GA [18]—has been applied to the investigation of a range of materials. In the perovskites, such as SrSnO${}_{3}$ [21] and SrTiO${}_{3}$ [22], it appears that rigid-unit motion is generally less significant than it is in the framework silicates. This is understandable inasmuch as the octahedra in perovskites have more bonding constraints, with each being bonded to six neighbours rather than to four in the case of tetrahedral frameworks. The geometric analysis still provides useful information on the prevalence of octahedral tilting in the framework.

A particularly interesting property of framework materials is the possibility of negative coefficients of thermal expansion (NCTE). Geometric analysis of structural models of zirconium tungstate, a cubic mineral displaying isotropic NCTE, confirms the significance of polyhedral rotations in generating NCTE [23]. By maximising the scope for such rotational motion in a framework, NCTE can be produced on a “colossal” scale [24].

### 4.2. Framework Flexibility and Motion

A particular characteristic of flexible framework structures, visible in the geometric analysis of dynamic disorder, is the capacity for substantial local rearrangements of the structure [25]. This is achieved by cooperative rotational modes involving many polyhedra. As a result, interatomic distances—for example, O–O distances defining a channel radius or pore aperture—can vary by amounts on the order of an Ångstrom without any great energetic penalty. This flexibility must be taken into account when considering interstitial ionic or molecular motion through a porous flexible framework [26,27]. Geometric simulation has been applied to investigate this flexibility in several systems.

**Figure 4:**
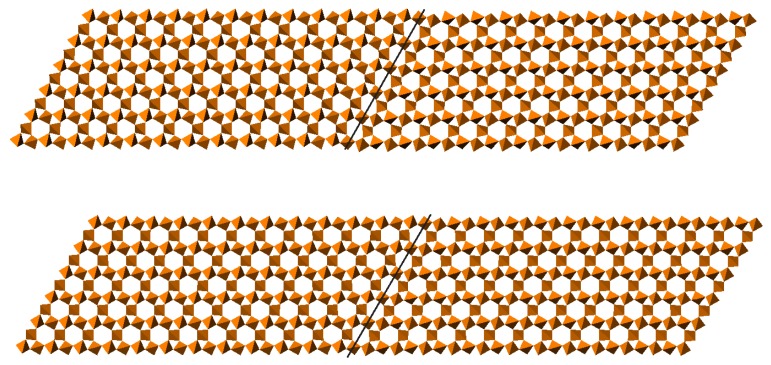
Structural models of domain walls in quartz, viewed down the crystallographic *c* axis (from [12]). The upper panel shows a geometrically relaxed configuration using α-quartz cell parameters, in which the domain wall (indicated by line) shows an elliptical channel profile. The lower panel shows a geometrically relaxed configuration using cell parameters from the incommensurate phase near the α–β phase transition; the domain wall is less distinct.

In quartz, channels run through the structure parallel to the crystallographic *c* axis. While in β-quartz the average channel profile is hexagonal, in α-quartz the hexagonal symmetry is broken. Domain walls occur between regions of the structure in which this symmetry breaking has occurred along different directions. In α-quartz the domain wall is a well-defined region, one channel wide, where the channels have a highly distorted elliptical profile. Geometric simulation of very large structural models [12] has shown how these well-defined domain walls become less distinct in an incommensurate phase just before the transition to the β phase (see Figure 4).

Framework flexibility is particularly relevant to the calculation of activation energies for ionic motion along channels. For the motion of Li${}^{+}$ ions through quartz channels, calculations based on the static average structure give misleadingly high activation energies; it appears that the Li${}^{+}$ ion has a well-defined preference for occupying certain sites along the channel and an activation energy is required to allow hopping from one site to the next. We have investigated this system using a combination of geometric simulation for framework relaxation with a Buckingham potential and localised electrostatic interaction to describe the interaction of the Li${}^{+}$ ion with the framework [11,12]. These simulations showed that the framework accommodates flexibly to the presence of the ion, so that motion along the channel is possible without a large activation energy. The results of the geometric simulation were corroborated by full potential calculations using GULP, and the resulting model of ionic motion successfully accounts for experimental data from dielectric spectroscopy and transport measurement on Li-doped quartz.

Framework flexibility also appears in the framework response to defects, such as the substitution of an aluminium for a silicon atom in an SiO${}_{2}$ framework. In addition to the electrostatic effects of the substitution, the longer Al–O bond length of 1.75 Å introduces strain into the framework. However, geometric analysis of defect simulations indicate [28] that cooperative motion in the framework allows “strain screening”; polyhedral distortion decreases rapidly with increasing distance from the defect site, as the framework adapts by rotations rather than distortions of the polyhedral units.

### 4.3. Compression Mechanisms in Zeolites

As geometric simulation brings out the flexibility intrinsic to the framework, it can usefully be applied to the study of compression mechanisms in framework structures, especially the aluminosilicate zeolites. Diffraction experiments on single-crystal or powder samples under compression (e.g., in a diamond-anvil cell) can provide cell parameters and hence pressure-volume (*P*-*V*) data, but may not always provide enough detail for structural refinement. A geometric simulation using the ambient-pressure framework topology and experimental cell parameters is a simple method to investigate the framework response to compression. As the cell parameters are altered, the polyhedra of the framework respond by rotation.

Studies on the edingtonite (**EDI**) [29] and levyne (**LEV**) [30] have revealed subtle connections between compression of the unit cell and the collective response of the framework. For example, in the levyne framework **LEV**, a form of “internal auxetic” effect couples compression of the cell to variations in channel profile in a non-obvious way—a uniform contraction of a channel profile is produced by a non-uniform compression of the cell.

### 4.4. Generation of Hypothetical Zeolites

Geometric analysis has been applied in a study of real and hypothetical 4-coordinated network structures representing zeolites and AlPOs [31], identifying limits on the permissible tetrahedral distortion if a framework is to be realisable physically. A key finding was that most hypothetical frameworks displayed much greater distortion of tetrahedral units than frameworks which are found experimentally to exist; this distinction had not been detected by considering the framework energy.

A current approach to the systematic generation of hypothetical zeolite structures is Symmetry-Constrained Intersite Bonding Search (SCIBS) [32,33]. The method proceeds from the generation of a topologically 4-coordinated network by symmetry, through its decoration with tetrahedral-centre T atoms and with vertex O atoms, and simulated annealing with a simple cost function [34], in order to generate a tetrahedral network which might represent a zeolite. The inclusion of a geometric simulation phase in the process proved to be effective in identifying at an early stage networks that were unable to attain tetrahedral geometry—for example if the TO${}_{4}$ groups were trapped in a square-planar geometry. The elimination of such unfeasible candidates saves considerable computational effort downstream in the simulated annealing process, giving an order of magnitude improvement in the rate of framework generation [35].

### 4.5. Application to Manganites: Modelling of Jahn–Teller Distortion

**Figure 5:**
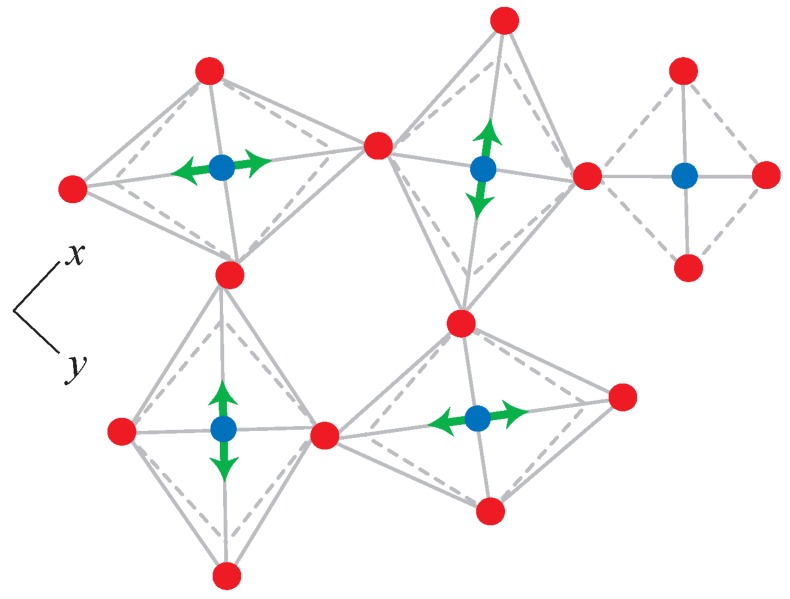
Use of regular (dashed outline) and Jahn–Teller distorted (solid outline) octahedral templates to model a manganite perovskite framework. From [36].

An interesting application of the method to an octahedral system is found in the study of the manganite perovskites. These systems can contain both regular octahedra centred on an Mn atom in the +4 oxidation state, and Jahn–Teller (JT) distorted polyhedra centred on Mn atoms in the +3 oxidation state. The latter contain four bonds in a square planar arrangement with a typical Mn–O length of 1.94 Å and two extended bonds perpendicular to the plane with a typical Mn–O length of 2.16 Å. JT-distorted octahedra can be represented in the template system by defining a privileged direction or “moment” for each octahedron and extending the bonds lying along that direction. The regular octahedra are represented as normal, as in Section 3. This use of templates is illustrated in Figure 5.

An investigation of LaMnO${}_{3}$ structures, using various user-defined patterns of moments, produced an intriguing result. For certain combinations of supercell parameters and moment distributions, the geometric simulation gives rise to “stripes”, a few polyhedra wide, generated by a pattern of variations in the polyhedral orientations [37]. Such stripe patterns, when observed experimentally, are conventionally attributed to variations in charge or oxidation states, whereas geometric simulation results suggest that ordered orientational variations may also be a cause.

Geometric templates also proved useful as constraints in a Reverse Monte Carlo (RMC) investigation of LaMnO${}_{3}$ based on neutron time-of-flight scattering data [36]. At high temperatures there is an apparent clash between the long-range average structure of LaMnO${}_{3}$, obtained from diffraction and crystal structure refinement, and local probes, in particular Pair Distribution Function (PDF) data [38]. Local structure indicates the persistence of JT distortion at high temperatures whereas the average structure indicates that the octahedra have become geometrically regular.

RMC modelling of a supercell structural model was attempted in order to generate structures consistent with both the long-range and local structural information. Such modelling must include constraints to ensure that the bonding geometry remains rational, so as not to generate unphysical structures. However, since the distribution of JT moments was to be an output of the modelling, it was important that the bonding constraints should not bias the presence or distribution of JT distortion. This was achieved using geometric templates in either regular or JT-distorted forms. Which template was applied to a given polyhedron was determined by its axial ratio in the model. Each attempted atomic move in the RMC simulation was followed by an accept/reject decision based on the mismatch between atoms and templates, with no such mismatch being allowed to exceed a threshold of 0.3 Å. The threshold allowed sufficient variation in the polyhedral geometry that a group of atoms could change from being considered JT-distorted to being considered approximately regular and back again.

This research successfully produced models for LaMnO${}_{3}$ which reflected both local and average structural data. The ordering of JT-distorted polyhedra provided a quadrupolar order parameter describing the apparent JT phase transition in LaMnO${}_{3}$. A matching study [9] of CaMnO${}_{3}$, in which JT distortion is absent in both the local and average structure, provided a “negative control”, demonstrating that the geometric constraints allowed the distribution of JT distortion to be driven by the experimental data.

### 4.6. The Flexibility Window of Zeolites

The discovery of the “flexibility window”, a geometric property of zeolite frameworks, is perhaps the most striking result to follow from geometric simulation to date [39,40]. A zeolite framework structure, determined experimentally from crystallographic refinement or simulated using interatomic potentials, always displays small variations of the atomic geometry in each tetrahedral unit away from the geometric tetrahedral ideal. From the point of view of geometric simulation, this immediately leads to a question: is it possible *in principle* for all tetrahedra to be made ideal? Or are distortions of the tetrahedral geometry inevitable, given the framework topology and the unit cell parameters of the structure?

This question can be answered using geometric simulation. If the tetrahedra can be made ideal, the structure can be geometrically relaxed until the residual mismatch between atom and template vertices is reduced to less than a small tolerance (we require that neither the bond-bending nor the bond-stretching distortion of any polyhedron should be greater than 0.001 Å). In this case we declare the structure fully geometrically relaxed, as the tetrahedra have reached ideal geometry. If the tetrahedra cannot be made ideal, the geometric relaxation stops with a residual mismatch exceeding the tolerance; the structure is not fully relaxed. We could also say that in the first case the geometric simulation produces a “stress-free” framework while in the second case there is an intrinsic stress in the framework.

**Figure 6:**
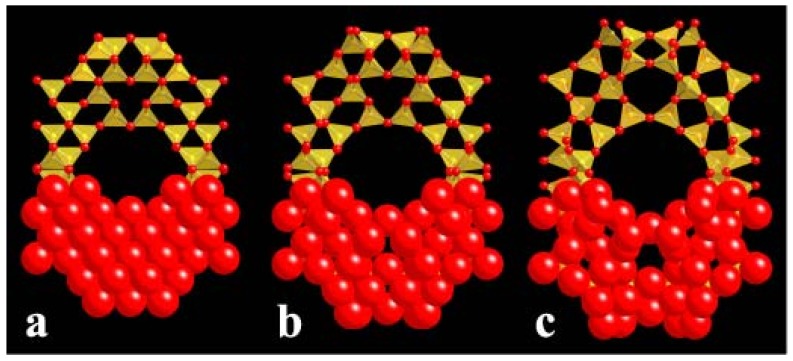
Faujasite (**FAU**) framework during an exploration by geometric simulation of the flexibility window, from the high-density limit at left to the low-density, expanded limit at right. The upper half of the framework is shown in a polyhedral view while the lower half is shown in a space-filling view to emphasise the significance of the oxygen atomic radii. From [39].

The striking result that emerges is that, for those zeolite frameworks which are known to exist in nature (either as minerals or through chemical synthesis), the framework can be geometrically relaxed so that the tetrahedra become geometrically ideal. Furthermore, this relaxation can be achieved over a range of densities. This leads to the definition of the *flexibility window* as the range of densities over which the tetrahedra of the framework can be made geometrically ideal. 196 of the 197 currently known zeolite framework types display a flexibility window [15]. The window is limited on the high-density side by collisions among oxygen atoms, and on the low-density side by tension in the T–O bonds (though not, typically, by an T–O–T angle of $180^{\circ }$), as illustrated in Figure 6. A curious feature is that the observed densities of zeolites under ambient conditions appear to lie towards the low-density edge of the window, indicating that zeolite frameworks are expanded structures [39].

In principle the flexibility window of a framework is defined in the six-dimensional space of the crystallographic cell parameters (a,b,c,α,β,γ). Investigations thus far have been restricted to lower dimensionality (1, 2 or 3) [15,16].

The real importance of the flexibility window is that, although it is a typical property of existing zeolite frameworks, it is a very rare property among hypothetical zeolite frameworks, even those calculated to have low framework energies using interatomic potentials [16,39]. It is more typical for hypothetical frameworks to display large intrinsic distortions of the tetrahedra. This suggests that the flexibility window may be a key criterion in the selection of hypothetical frameworks as candidates for synthesis [40].

Although the flexibility window is defined geometrically, it it also linked to the physics of zeolite frameworks. We can imagine dividing the interatomic interactions in a zeolite structure into two components, $U_{local}$ and $U_{nonlocal}$. $U_{local}$ contains those interactions which favour ideal tetrahedral geometry and steric exclusion, while $U_{nonlocal}$ contains all other interactions, including long-range dispersion and electrostatic interactions. The geometric simulation effectively models $U_{local}$ while neglecting $U_{nonlocal}$.

If the structure lies within its flexibility window, that is, it can be fully geometrically relaxed, then it can reach the minimum of $U_{local}$. If we now introduce $U_{nonlocal}$ as well, there will be some distortion of the TO${}_{4}$ units from the tetrahedral geometric ideal. This distortion represents variation around a minimum and so its energy penalty in the $U_{local}$ terms will be small and second-order in the size of the distortion. If on the other hand the structure lies outside its flexibility window, it cannot reach the minimum of $U_{local}$. Any further distortion of the framework will come at an energy penalty which is large and first-order in the size of the distortion. We therefore expect the structure to behave differently in, for example, the interactions between the framework and channel contents, depending on whether it lies inside or outside its flexibility window. This insight has led to further studies identifying links between the flexibility window and the physical properties of zeolites, especially in their response to pressure, as we shall see in Section 4.7.

### 4.7. Phase Transitions in Zeolites

Since the high-density edge of the flexibility window is defined by clashes among vertex oxygen atoms, we expect that the framework will change its behaviour under compression if it approaches this edge of the window. We have therefore begun to investigate the occurrence of phase transitions in zeolites under pressure, using geometric simulation and the flexibility window concept to interpret experimental data.

Cubic analcime displays a phase transition at a relatively low pressure (∼1 GPa) from a highly symmetric cubic form to a minimally symmetric triclinic form; this transition is also associated with an “anomalous” softening, that is, the high-pressure form displays a lower bulk modulus than the ambient-pressure form. Using geometric simulation we have shown that this transition occurs when the structure reaches the edge of its cubic flexibility window [41]. This insight extends to other minerals with the **ANA** framework—leucite, pollucite and wairakite [42]—and in wairakite, Al/Si ordering in the framework is significant [43].

### 4.8. Application to Proteins

Template-based geometric simulation has also been applied in biophysics as a method for simulating flexible motion in proteins [44]. The “FRODA” approach (Framework Rigidity Optimised Dynamic Algorithm) makes use of templates to represent relatively rigid groups of atoms in a protein structure. The flexibility of the protein can then be explored within the constraints imposed by steric exclusion and the templates, at a significantly lower computational cost than conventional molecular dynamics. FRODA [44] was originally implemented as a module within the “FIRST” rigidity analysis software [45] and template-based geometric simulation is also used in the recent FRODAN [46] and NMsim [47] methods. The combination of rigidity analysis, template-based geometric simulation and elastic network modelling is particularly promising for rapid simulation of flexible motion [48].

**Figure 7:**
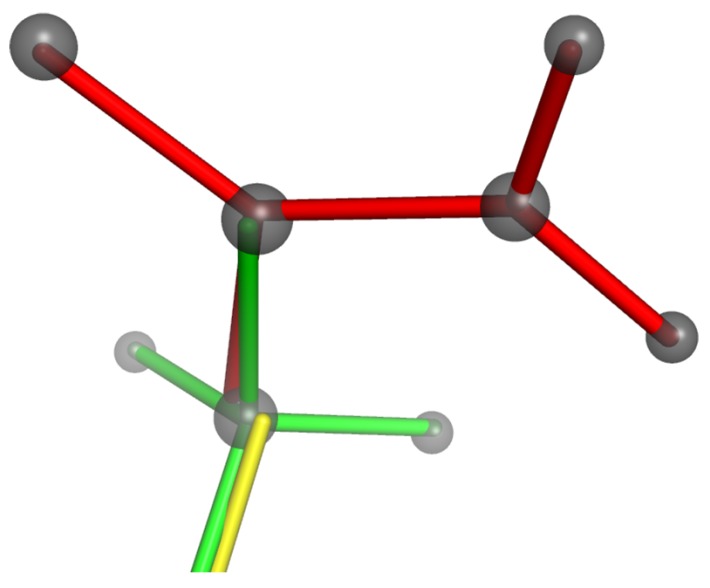
Use of overlapping templates (shown as red, green and yellow sticks) as constraints in the simulation of proteins. The tethering of atoms (grey) to these overlapping templates constrains interatomic bond lengths and angles but allows dihedral angles to vary.

There are two main differences between the use of templates for simulations of mineral frameworks and their use for proteins. The first difference is that, in mineral frameworks, the templates represent a central atom and its nearest-neighbour vertex atoms. In proteins, a single template can cover larger groups of atoms, identified using rigidity analysis; the simulation therefore includes templates with a wide range of sizes and shapes, ranging from individual methyl groups to entire domains including multiple secondary structure units. The second difference is that, in mineral frameworks, the bridging angles between polyhedra are treated as variable. In proteins, by contrast, flexibility is permitted by variation of the dihedral angles. This is represented by allowing templates to overlap along bonds where the dihedral angle is variable, as illustrated in Figure 7. Two templates are thus constrained to have a bond vector in common.

## 5. Availability of Geometric Simulation Codes

GASP code is available without charge on request to academic users, who should contact the corresponding authors, and may be freely used and modified for research.
